# Preliminary experience with controlled hypothermic storage of donor heart in combined heart transplantation

**DOI:** 10.1016/j.jhlto.2026.100707

**Published:** 2026-10-14

**Authors:** Salem T. Argaw, Lorenzo Lora Ronco, Anouk Frering, Martin Kloeckner, Guillaume Coutance, Audrey Coilly, Eric Vibert, Renaud Snanoudj, Thibaut Genty, Paul Achouh, Emre Belli, Julien Guihaire

**Affiliations:** a Adult Cardiac Surgery and Transplantation, Marie Lannelongue Hospital, Groupe Hospitalier Paris Saint Joseph, Le Plessis Robinson, France; b Adult Cardiac Surgery and Transplantation, Hôpital Européen Georges-Pompidou, Paris, France; c Department of Adult Cardiac Disease, Marie Lannelongue Hospital, Groupe Hospitalier Paris Saint Joseph, Le Plessis Robinson, France; d Hepatobiliary Centre, Paul-Brousse Hospital, AP-HP, Paris-Saclay University, INSERM U1193, Villejuif, France; e Nephrology-Dialysis-Transplantation, Bicêtre Hospital, AP-HP, LeKremlin-Bicêtre, France; INSERM U1184, Le Kremlin-Bicêtre, France; f Adult Intensive Care Unit, Marie Lannelongue Hospital, Groupe Hospitalier Paris Saint Joseph, Le Plessis Robinson, France; g Inserm UMR_S1358, Medical School, University of Paris Saclay, Le Kremlin Bicêtre, France; h Congenital Cardiac Surgery and Transplantation, Marie Lannelongue Hospital, Groupe Hospitalier Paris Saint Joseph, Le Plessis Robinson, France

**Keywords:** combined heart transplantation, ice cold storage, heart-liver transplant, heart-kidney transplant, controlled hypothermic storage

## Abstract

While studies have demonstrated improved clinical outcomes with the use of controlled hypothermic storage (CHS) devices in isolated heart transplantation, it has not been evaluated in combined heart transplantation. In this small retrospective study (n=17), we evaluate clinical outcomes after combined heart-liver and heart-kidney transplantation in recipients of donor hearts preserved with conventional ice cold storage (ICS, n=10) versus CHS (n=7). The study found severe early graft dysfunction (EGD) occurred more frequently in the ICS cohort when compared to the CHS cohort (70% vs 0%, respectively). In addition, the CHS cohort had shorter median intensive care unit length of stay (7 days compared to 15.5 days, respectively) as well as fewer post-transplant complications, including sepsis (60% in ICS and 28.6% in CHS), pneumonia (40% in ICS and 14.3% in CHS), stroke (30% in ICS and 0% in CHS), and acute kidney injury requiring dialysis (60% in ICS and 28.6% in CHS). These preliminary findings suggest CHS may improve outcomes in combined heart transplantation.

Several studies have compared conventional ice cold storage (ICS) with controlled hypothermic storage (CHS) for donor heart preservation using data from the Global Utilization And Registry Database for Improved heArt preservatioN (GUARDIAN-Heart) registry. CHS aims to preserve the cooling benefits of reduced myocardial metabolic activity at hypothermic temperatures while minimizing the risk of freezing injury and associated cellular damage that may contribute to graft dysfunction^1^. When comparing CHS to ICS, recent studies have observed lower rates of severe primary graft dysfunction, less mechanical circulatory support use, and reduced hospital stays^2^, ^3^. This decrease in resource utilization and hospital costs allows for reallocation of healthcare resources, potentially increasing capacity for a greater number of complex cases. While the use of CHS in isolated heart transplantation has been well-documented, there have been no reports on its utility in combined heart transplantation. For example, while the recently published COMBI study provided comprehensive survival data for combined heart and other organ transplantation in Spain, the impact of donor heart preservation strategy was not evaluated^4^. This study evaluates outcomes after combined heart-liver (HL) and heart-kidney (HK) transplantation in recipients of donor hearts preserved with conventional ICS versus CHS.

Following ethical approval by the Institutional Review Board of the French Society of Thoracic and Cardiovascular Surgery (IRB00012919), a retrospective analysis was conducted. Adult patients who underwent a combined HL or HK transplant at Marie Lannelongue Hospital (Le Plessis Robinson, France) or Georges Pompidou European Hospital (Paris, France) between January 1st, 2020, and May 1st, 2026, were included. In accordance with national and hospital policy, patients were informed of the study details and offered a chance to decline participation. All recipients underwent single-stage combined transplantation using grafts from the same donor, with implantation of the cardiac allograft performed prior to implantation of the second organ during the same operation. SherpaPak Cardiac Transport System (Paragonix Technologies, Waltham, MA, USA), a controlled hypothermic preservation system designed to maintain donor hearts within a target temperature range of 4–8°C while immersed in cold preservation solution, was used for CHS. In the ICS cohort, donor organs were stored in sterile preservation solution and transported in an insulated container including four reusable refrigerant gel packs (Vitalpack EVO, E3 Cortex, Plailly, France). Cardiac grafts were preferentially preserved using CHS following the device’s implementation at both institutions in 2023 (supplementary figure 1); prior to this, conventional static cold storage was used. Livers were preserved using static cold storage, while kidneys were preserved using hypothermic machine perfusion. The primary endpoint was severe early graft dysfunction (EGD) of the cardiac allograft defined as graft dysfunction of unknown etiology occurring in the first 24hrs and requiring mechanical circulatory support, in accordance with the new ISHLT consensus summary^5^. Secondary endpoints included postoperative extracorporeal membrane oxygenation (ECMO) utilization, duration of mechanical ventilation, incidence of post-transplant dialysis, incidence of acute cardiac rejection, incidence of early kidney or liver allograft failure, intensive care unit (ICU) length of stay (LOS), hospital LOS, and mortality. In patients requiring ECMO, support was initiated intraoperatively following cardiac allograft implantation and prior to implantation of the second organ. Ischemic time is reported as total ischemic time because separate cold and warm ischemic times were not available. Delayed graft function of the kidney allograft was defined as the need for renal replacement therapy within the first post-transplant week^6^. Early allograft dysfunction of the liver was defined according to the definifition provided by Olthoff et al.: bilirubin ≥10 mg/dL or INR ≥1.6 on post-operative day 7, or ALT/AST >2000 IU/L within the first 7 days^7^. Categorical variables are presented as counts and percentages, and continuous variables as median (IQR). Given the limited sample size, analyses were descriptive in nature.

A total of 17 patients were included, 10 of which were in the ICS cohort and 7 in the CHS cohort (Table 1). Donor characteristics were relatively balanced between the two cohorts and median total ischemic time for the heart was 224 min (204 in the ICS cohort and 228 in the CHS cohort, Table 2). Recipients in the CHS cohort generally exhibited a higher body mass index (BMI), lower left ventricular ejection fraction (LVEF), higher incidence of prior cardiac surgery, and longer waitlist times than those in the ICS cohort. Of the 8 patients undergoing redo sternotomy, 7 (3 in the ICS cohort and 4 in the CHS cohort) had undergone their initial operation for congenital heart disease (CHD). The 8th patient had a history of valve surgery. Six of the 8 undergoing redo sternotomy were HL transplant recipients, all of whom had previously undergone surgery for CHD. The remaining 2 occurred in HK recipients and included one patient with prior CHD surgery and one with prior valve surgery.

**Table 1:** Patient-Level Characteristics of Heart-Liver and Heart-Kidney Transplant Patients using ICS Versus CHS

**Pt**	**Surgery**	**Instit-ution**	**Age (yrs) / Sex**	**Tx Date**	**Cardiac graftpreservation method**	Severe EGD*(ECMO/PGD)	**Pre-Txcreatinine (µmol/L)**	**Pre-Tx eGFR**^**+**^**(mL/min/1.73m²)**	**Heart isch.time (min)**	**Kidneyisch. Time (min)**	**Liver isch.time (min)**	**Renal allograft outcome**	**Hepatic allograft outcome**	**Mortality**
**1**	H-L	HML	34 / M	03/2021	ICS	Yes (ECMO 7d)	69	—	215	—	606	Post-op RRT	NSF	Alive
**2**	H-L	HML	44 / F	06/2022	ICS	Yes (ECMO 3d)	86	—	192	—	594	AKI KDIGO 3; Post-op RRT	NSF	Deceased — refractory septic shock
**3**	H-K	HML	62 / M	06/2022	ICS	Yes (ECMO 4d)	766	6	147	610	—	AKI	—	Alive
**4**	H-K	HML	31 / M	07/2022	ICS	No	774	8	130	400	—	NSF	—	Alive
**5**	H-L	HEGP	43 / F	04/2023	ICS	Yes (ECMO 6d)	91	—	261	—	660	AKI KDIGO 3	Early liver graft failure	Deceased — stroke
**6**	H-L	HML	47 / F	05/2023	ICS	No	N/A	—	257	—	519	AKI KDIGO 3; Post-op RRT	NSF	Alive
**7**	H-L	HML	64 / M	05/2023	ICS	Yes (ECMO 2d)	255	—	235	—	630	AKI KDIGO 3; Post-op RRT	NSF	Alive
**8**	H-K	HEGP	63 / F	06/2023	ICS	Yes (ECMO 8d)	600	6	233	742	—	AKI; Post-op RRT; early kidney graft failure	—	Alive
**9**	H-K	HML	54 / M	06/2023	CHS	No	251	26	249	780	—	AKI; Post-op RRT	—	Alive
**10**	H-L	HML	48 / M	07/2023	ICS	No	100	—	180	—	443	AKI KDIGO 3; Post-op RRT	NSF	Alive
**11**	H-K	HEGP	41 / F	11/2023	ICS	Yes (ECMO 1d)	519	9	140	481	—	AKI	—	Alive
**12**	H-K	HML	59 / M	06/2024	CHS	No	230	28	234	N/A	—	NSF	—	Alive
**13**	H-L	HML	36 / F	07/2024	CHS	No	88	—	132	—	N/A	NSF	NSF	Alive
**14**	H-K	HEGP	55 / F	08/2024	CHS	No	105	54	161	563	—	NSF	—	Alive
**15**	H-L	HML	30 / F	01/2025	CHS	No	54	—	224	—	500	NSF	NSF	Alive
**16**	H-L	HML	47 / F	04/2025	CHS	No	N/A	—	231	—	525	AKI KDIGO 3; Post-op RRT	NSF	Deceased — refractory septic shock
**17**	H-K	HML	21 / M	08/2025	CHS	No	178	47	228	697	—	NSF	—	Alive

CHS, controlled hypothermic storage; ICS, ice cold storage; Pt, patient; tx, transplant; EGD, early graft dysfunction; ECMO, extracorporeal membrane oxygenation with duration of support; H-L, combined heart-liver transplantation; H-K, combined heart-kidney transplantation; HEGP, Hôpital Européen Georges-Pompidou; HML, Hôpital Marie Lannelongue; eGFR, estimated glomerular filtration rate; Heart / Kidney / Liver isch. Time, total ischemic time in minutes of the corresponding graft; AKI, acute kidney injury in intensive care (KDIGO classification); RRT, renal replacement therapy; NSF, nothing significant to report / no complication reported on this domain

—: not applicable (organ not transplanted in this patient)

*Severe EGD defined as graft dysfunction of unknown etiology occurring in the first 24hrs and requiring mechanical circulatory support, in accordance with the new ISHLT consensus summary ^5^.

+eGFR calculated using the CKD-EPI 2021 equation (race-free) from pre-transplant creatinine; reported only for the heart-kidney subgroup.

**Table 2:** Baseline Patient Characteristics and Post-transplant Outcomes of Heart-Liver and Heart-Kidney Transplant Patients using ICS Versus CHS

	Total (n=17)	ICS(n=10)	CHS(n=7)
Donor characteristics			
Age (years)	47 (33.5–54.5)	50 (34–55)	46 (32–54)
BMI (kg/m^2^)	23.2 (22.0–25.6)	23.2 (22.0–24.4)	22.0 (21.8–28.0)
LVEF (%)	65 (62.5–70)	65 (60–70)	65 (65–70)
F/M mismatch	3 (17.6%)	2 (20%)	1 (14.3%)
PHM ratio	1.01 (0.94–1.07)	1.02 (0.99–1.07)	0.94 (0.89–1.07)
Most undersized PHM (<-15%)	2 (11.8%)	1 (10%)	1 (14.3%)
Distance to organ (km)	220 (20–438)	230 (20–385)	220 (20–490)
Total ischemic time (min)	224 (154–234.5)	204 (147–235)	228 (161–234)
CHS min temp (°C)	-	-	6.3 (5.3–7.3)
CHS max temp (°C)	-	-	8.0 (6.6–9.3)
Recipient characteristics			
Age (years)	47 (35–57)	45.5 (41–62)	47 (30–55)
BMI (kg/m^2^)	23.1 (19.7–25.8)	22.9 (20.0–23.9)	27.3 (19.2–29.7)
LVEF (%)	50 (23.0–57.5)	50 (25–60)	37 (21–55)
Prior cardiac surgery	8 (47.1%)	3 (30%)	5 (71.4%)
Wait list time (days)	117 (52–177)	96 (41–359)	157 (50–190)
Durable VAD	0	0	0
Temporary ECMO	0	0	0
Combined H-L	9 (52.9%)	6 (60%)	3 (42.9%)
Combined H-K	8 (47.1%)	4 (40%)	4 (57.1%)
Post-transplant outcomes			
Severe EGD*	7 (41.2%)	7 (70%)	0
Duration of ECMO (days)	4 (2–7)	4 (2–7)	-
Post-tx cardiac arrest	2 (11.8%)	1 (10%)	1 (14.3%)
Duration of mechanical ventilation (hrs)	18 (7.5–120)	15.5 (6–120)	21 (9–24)
Sepsis	8 (47.1%)	6 (60%)	2 (28.6%)
Pneumonia	5 (29.4%)	4 (40%)	1 (14.3%)
Stroke	3 (17.6%)	3 (30%)	0
AKI requiring post-tx dialysis	8 (47.1%)	6 (60%)	2 (28.6%)
Acute cardiac rejection^a^	4 (23.5%)	2 (20%)	2 (28.6%)
Early kidney failure**	2/8 (25%)	1/4 (25%)	1/4 (25%)
Early liver failure	2/9 (22.2%)	1/6 (16.7%)	1/3 (33.3%)
ICU LOS (days)	9 (6–22.5)	15.5 (8–25)	7 (5–12)
Hospital LOS (days)	29 (22–45)	27.5 (18–58.5)	34 (28–38)
30-day mortality	1 (5.9%)	1 (10%))	0
Overall mortality	3 (17.6%)	2 (20%)	1 (14.3%)

Results are described as median and IQR for continuous variables and counts/percentages for categorical variables.

ICS, ice cold storage; CHS, controlled hypothermic storage; BMI, body mass index; LVEF, left ventricular ejection fraction; F/M mismatch, female to male mismatch; PHM, predicted heart mass; VAD, ventricular assist device; ECMO, extracorporeal membrane oxygenation; Combined H-L, combined heart-liver transplant; Combined H-K, combined heart-kidney; tx, transplant; EGD, early graft dysfunction; AKI, acute kidney injury; ICU LOS, intensive care unit length of stay.

* Severe EGD was defined as graft dysfunction of unknown etiology occurring within the first 24 h and requiring mechanical circulatory support, in accordance with the new ISHLT consensus summary (5).

** Early kidney failure after kidney transplantation included the need for post-transplant dialysis.

a Acute cardiac rejection requiring ongoing treatment at 1year

Seven (4 HL and 3 HK recipients) of 10 in the ICS cohort met criteria for severe EGD due to post-transplant ECMO utilization, whereas no patients in the CHS cohort met this criterion (Table 2). Post-transplant complications, including sepsis, pneumonia, stroke, and acute kidney injury (AKI) requiring dialysis, were also observed more frequently in the ICS cohort. However, patients in the CHS cohort had a higher median duration of mechanical ventilation when compared to patients in the ICS cohort. Four patients total (2 in ICS, 20%, and 2 in CHS, 28.6%) experienced acute cardiac rejection. Two of 8 HK patients experienced early kidney allograft failure and required dialysis; however, there were no kidney allograft rejection or failure at 1 year among patients who reached the 1-year follow-up timepoint. Similarly, 2 of 9 HL patients experienced early liver allograft failure, but no liver allograft rejection or failure was seen at 1 year among patients who reached the 1-year follow-up timepoint. Although the median hospital length of stay was longer in the CHS cohort, the median intensive care unit LOS was shorter at 7 days, compared with 15.5 days in the ICS cohort. Three patients (all HL transplant recipients) died during the study period, including two in the ICS cohort and one in the CHS cohort (Table 3). Causes of death included septic shock (n=2) and stroke (n=1).

**Table 3:** Characteristics of Mortality Cases

	Total deaths(n=3)
ICS	2 (66.7%)
CHS	1 (33.3%)
Heart-liver recipients	3 (100%)
Heart-kidney recipients	0
Prior cardiac surgery	2 (66.7%)
Total cardiac ischemic time (min)	231 (192–261)
Severe EGD	2 (66.7%)
Duration of mechanical ventilation (hrs)	504 (6–600)
Sepsis	2 (66.7%)
Pneumonia	2 (66.7%)
Stroke	1 (33.3%)
ICU LOS (days)	25 (6−37)
Acute cardiac rejection	1 (33.3%)
Early liver failure	2 (66.7%)

Results are described as median and range for continuous variables and counts/percentages for categorical variables.

ICS, ice cold storage; CHS, controlled hypothermic storage; EGD, early graft dysfunction; ICU, intensive care unit; LOS, length of stay.

This study observed that severe EGD occurred more frequently in the ICS cohort despite a greater burden of baseline risk factors, including higher BMI, lower LVEF, and a higher prevalence of prior cardiac surgery in the CHS cohort. The CHS cohort had shorter median ICU LOS and fewer post-transplant complications, including sepsis, pneumonia, stroke, and AKI requiring dialysis. These findings are consistent with previously reported benefits of CHS in isolated heart transplantation and suggest that similar observations may extend to combined heart transplantation^2^, ^8^.

Median follow-up was approximately 35.5 months in the ICS group and 21.0 months in the CHS group. The 30-day mortality and overall mortality observed in this study were 5.9% and 17.6%, respectively. All deaths occurred among HL transplant recipients (3/9) and 2 of 3 patients who died were in the ICS cohort. Two of 3 deaths also occurred in patients with severe EGD. While interpretation is limited by the small sample size and differing follow up times, the 30-day mortality among HL recipients (11.1%) was in the range of that reported for HL recipients in the COMBI study (12.5%)^4^. However, previous studies, such as Rizvi et al.’s 2019 meta-analysis, have reported 30-day mortality as low as 5% among HL recipients^9^.

This study has several limitations such as its retrospective design and small sample size which limit our ability to make definitive conclusions on the effects of preservation strategies in combined heart transplants. Additionally, the study is subject to a potential era effect because ICS was used predominantly earlier in the study period and CHS later. The observed associations between preservation strategy and postoperative outcomes may therefore be confounded by temporal changes in institutional experience, perioperative management practices, and differing follow up times. Moreover, the ICS cohort had a greater proportion of the HL patients which had a higher rate of complications and mortality. Lastly, due to broad referral patterns, access to long-term follow-up and survival data was limited. Larger, multicenter studies with extended follow-up are needed to further characterize these findings.

## Financial support

This work is part of the TREASURE research program (ANR-25-APTR-0001), funded under the France 2030 plan.

## Patient consent

Authors confirm that appropriate patient consent to publish this case report was received.

## CRediT authorship contribution statement

Julien Guihaire: Conceptualization, Methodology, Writing - review and editing. Emre Belli: Conceptualization, Methodology, Writing - review and editing. Salem Argaw: Writing - Original draft, Visualization. Lora Ronco Lorenzo: Investigation, Writing - review and editing. Anouk Frering: Investigation, Writing - review and editing. Martin Kloeckner: Writing - review and editing, Supervision. Guillaume Coutance: Writing - review and editing, Supervision. Audrey Coilly: Writing - review and editing, Supervision. Eric Vibert: Writing - review and editing, Supervision. Renaud Snanoudj: Writing - review and editing, Supervision. Thibaut Genty: Writing - review and editing, Supervision. Paul Achouh: Writing - review and editing, Supervision.

## Conflicts of Interest statement

The authors declare that they have no known competing financial interests or personal relationships that could have appeared to influence the work reported in this paper.

## Footnotes

Appendix A Supplemental data associated with this article can be found in the online version at doi:10.1016/j.jhlto.2026.100707.
