# Supplementary material for: Preliminary experience with controlled hypothermic storage of donor heart in combined heart transplantation
Source: JHLT Open. 2026 Oct 14;15:100707. doi: 10.1016/j.jhlto.2026.100707 (PMC13743936; doi:10.1016/j.jhlto.2026.100707)
Supplement: Supplementary file 1 [file mmc1.docx]

**Supplementary figure 1: Schematic representation of cardiac graft preservation using the SherpaPak Cardiac Transport System (SCTS).**

Following donor heart explantation, the cardiac allograft is secured to the SCTS heart connector via the aorta and suspended within the inner canister. The heart is immersed in cold preservation solution, with direct monitoring of preservation-solution temperature using an internal temperature probe. The inner canister is placed within the outer canister and incorporated into the insulated transport shipper with SherpaCool® phase-change cooling elements. The system provides static hypothermic preservation of the cardiac allograft during transport, with temperature data monitored and recorded throughout the preservation period.
